# Glycophosphopeptical AM3 Food Supplement: A Potential Adjuvant in the Treatment and Vaccination of SARS-CoV-2

**DOI:** 10.3389/fimmu.2021.698672

**Published:** 2021-06-17

**Authors:** Diego Fernández-Lázaro, Cesar I. Fernandez-Lazaro, Juan Mielgo-Ayuso, David P. Adams, Juan Luis García Hernández, Jerónimo González-Bernal, Marcela González-Gross

**Affiliations:** ^1^Department of Cellular Biology, Histology and Pharmacology, Faculty of Health Sciences, University of Valladolid, Soria, Spain; ^2^Neurobiology Research Group, Faculty of Medicine, University of Valladolid, Valladolid, Spain; ^3^Department of Preventive Medicine and Public Health, School of Medicine, University of Navarra, Navarra Institute for Health Research (IdiSNA), Pamplona, Spain; ^4^Department of Health Sciences, Faculty of Health Sciences, University of Burgos, Burgos, Spain; ^5^Nutrition, Exercise and Healthy Lifestyle Research Group (ImFINE) Research Group, Department of Health and Human Performance, Faculty of Physical Activity and Sport Sciences-National Institute of Physical Education (INEF), Polytechnic University of Madrid, Madrid, Spain; ^6^Dual Enrollment Program, Point University, Savannah, GA, United States; ^7^Cancer Research Centre, University of Salamanca, Salamanca, Spain

**Keywords:** COVID-19, AM3, glycophosphopeptical, food supplement, immunonutrition, cytokines, muscular damage, vaccination

## Abstract

The world is currently experiencing the coronavirus disease 2019 (COVID-19) pandemic caused by Severe Acute Respiratory Syndrome-2 (SARS-CoV-2). Its global spread has resulted in millions of confirmed infections and deaths. While the global pandemic continues to grow, the availability of drugs to treat COVID-19 infections remains limited to supportive treatments. Moreover, the current speed of vaccination campaigns in many countries has been slow. Natural substrates with biological immunomodulatory activity, such as glucans, may represent an adjuvant therapeutic agent to treat SARS-CoV-2. AM3, a natural glycophosphopeptical, has previously been shown to effectively slow, with no side effects, the progression of infectious respiratory diseases by regulating effects on innate and adaptive immunity in experimental models. No clinical studies, however, exist on the use of AM3 in SARS-CoV-2 infected patients. This review aims to summarize the beneficial effects of AM3 on respiratory diseases, the inflammatory response, modulation of immune response, and attenuation of muscle. It will also discuss its potential effects as an immune system adjuvant for the treatment of COVID-19 infections and adjuvant for SARS-CoV-2 vaccination.

## Introduction

The world has experienced, and today continues experiencing, the COVID-19 pandemic caused by Severe Acute Respiratory Syndrome-2 (SARS-CoV-2). Its transmission probability has been estimated in the beginning of the pandemic in 1.4-2.5 (1) which has caused a rapid spread worldwide resulting in millions of deaths (2). SARS-CoV-2 infection causes a wide range of symptoms. While a sizable number of patients present flu-like symptoms, others develop a severe condition associated with respiratory distress and pneumonia (3). These cases are also characterized by Acute Respiratory Distress Syndrome (ARDS), renal failure, septic shock or multi-organ failure, conditions that generally require hospitalization, intensive care unit (ICU) admission, and/or mechanical ventilation (4) and a multisystem inflammatory syndrome (5). In relation to the spectrum of the COVID-19, 81% suffer from a mild illness, 14% require hospitalization, of which 6% suffer from serious illness, and 5% need admission to hospital and/or ventilation in the ICU. Although the case fatality rate varies by country, overall it is roughly 2% (6).

The current availability of drugs to treat COVID-19 infections remains limited to supportive treatments. These are the main methods of care such as supplemental oxygen and mechanical ventilator support in severe and critical cases (7). However, drugs such as antiparasitics, including antimalarial drugs based on *in vitro* or animal-model antiviral activity, antibiotics, broad-spectrum antivirals and other modern therapeutic agents have been reused (8). Clinical trials have focused on the antimalarial drugs chloroquine and hydroxychloroquine, the antibiotic drug azithromycin, and convalescent plasma transfusion (9). The knowledge of SARS-CoV-2 virology provides a considerable number of possible targets for antiviral drugs. However, there are still no conclusive data on the efficacy of antivirals such as Ribavirin, Oseltamivir, Favipiravir, and the anti-tumor drug Plitidepsin. Their substantial toxicity suggests that it has limited value for the treatment of COVID-19 (10). None of these treatments have been approved by any regulatory body yet. Still, Remdesivir is currently the most promising as it has been granted an Emergency Use Authorization by the United States Food and Drug Administration (FDA or USFDA) as it undergoes clinical trials Phase III. Remdesivir reduces the stay in intensive care, although without significant clinical effects (11). Another option is the use of several antivirals in combination (Lopinavir, Ritonavir, Ribavirin) associated with Interferon 1β which has been shown to reduce symptoms and reduce the temporality of the infective phases of COVID-19. However, the therapeutic regimen is complex and its availability in hospitals is limited (12).

Therefore, the temporal need to find an effective antiviral agent is critical, and except for Dexamethasone, has not been shown to be any effective treatment. Dexamethasone, a glucocorticosteroid, is able to reduce the body’s immune response which could modulate the COVID-19 multisystem inflammatory syndrome (13). This drug offers the most cost-effective treatment for COVID-19 which can reduce mortality by 20% in patients on oxygen therapy and by 30% in patients on automatic ventilation (14). It also has an anti-viral effect by binding to the hormone cortisol, stimulating the production of anti-inflammatory cytokines and inhibiting the secretion of pro-inflammatory cytokines that cause COVID-19 pneumonia (15).

An alternative approach to prevent COVID-19 infections is vaccination. This strategy represents the most effective prevention measure to help end the pandemic (16). More than 100 prototype vaccines against SARS-CoV-2 have been tested, and a limited number have been approved by the regulatory authorities. Briefly, COVID-19 vaccines aim to expose the body to an antigen that does not cause disease but, rather, sparks an immune response that blocks or destroys the virus in the face of an attempted infection (17). Such strategies can be categorized into: viral vaccines, attenuated viruses or inactivated viruses; vaccines containing the genetic instructions of a protein, nucleic acids in the form of DNA or RNA; vaccines with viral vectors, replicative vectors (attenuated measles) or non-replicative vectors adenovirus); and vaccines of a protein nature, by injecting subunits of COVID-19 structural proteins or viral structural particles that mimic the structure of SARS-CoV-2 (18). Currently, the process of vaccination against SARS-CoV-2 is slow among the majority of the countries, having vaccinated only 10% of the world’s population (19). As such, new modalities are needed to reduce the burden of SARS-CoV-2.

In this context, alleviation of symptoms or enhancement of the healing process by other bioactive molecules with health-promoting properties (e.g., essential nutrients, herbal extracts, herbal extracts, phytochemicals and nutraceuticals) may offer an alternative strategy when no effective pharmacological treatments are available (20). Another alternative may be the use of natural substrates, such as the glucans, a group of substrates with biological immunomodulatory activity (21). These immunomodulators are molecules that have been previously used in clinical medicine and have been shown to improve health outcomes (22).

We describe in this paper a hypothetical therapeutic option of the biological effects of glucans and, more specifically, of the natural glucan glycophosphopeptical AM3 against COVID-19. Based on previous research that has documented positive effects on respiratory diseases (23, 24), regulation on inflammatory response (25–27), modulation of immune response (25–29), attenuation of muscle (29–31) and lung damage (32). This manuscript discusses AM3 as a potential adjuvant therapeutic agent, which could play a role in the prophylaxis or amelioration of symptoms associated with COVID-19. We also describe the potential benefits of AM3 as an immune system adjuvant for the control of SARS-CoV-2 infections through immunoprophylaxis, based on previous studies (33, 34).

## Glycophosphopeptical AM3

Any foreign antigen such as an infectious agent stimulates the immune system to some degree. Such heightened activity is accompanied by an increased rate of metabolism. As a result, the body needs some substrates to induce the production of mediators and effectors of the immune response. Therefore, additional energy sources, which are substrates and molecules, which may come from dietary intake, are required for biosynthesis of immune mediators for optimal functioning of the immune system (35, 36). Some of these substrates are glucans, molecules that perform preliminary innate actions triggered by pathogen-associated molecules (37). Glucans are linear glucose homo-polysaccharides linked to β-(1→3) and β-(1→4). Differences in the linkages and branching influence the size of the molecule, its tertiary structure, electrical charge, conformation in solution (triple or single helix, or random spiral), and its solubility properties (38).

AM3 is a glycophosphopeptide composed of Candida utilis yeast phosphorylated glucomannan polysaccharide and Ricinus communis protein in a 5: 1 ratio (polysaccharide: protein). AM3 has been commercialized as an oral pharmaceutical product marketed as Immunoferon^®^ by Cantabria-Labs (Santander, Spain). The Spanish Agency for Food Safety and Nutrition (AESAN) categorizes AM3 as “Food Supplement”. The polysaccharide element of AM3 is a phosphoglucomannan-type B-glucan (GLPH-1; approximately 15 kDa), which contains repeating polysaccharides linear chains (10-40 repeats), with β(1→6) and β(1→2) links between and within the mannose and glucose residues in a ratio of 12: 1 (mannose: glucose). The protein element is a member of the 2S albumin family of proteins derived from RicC3 (27, 39). The protein fraction of RicC3 (12.0 kDa) consists of two subunits of different sizes that form a heterodimeric structure with very stable disulfide bridge bonds. Moreover, RicC3 has a five-helix bundle folded into a right-handed superhelix (40). In short, in AM3 the active ingredient is a 5:1 (w/w) mixture of polysaccharide and protein (AM3) (27).

The biopharmaceutical properties of AM3 allow it to produce an immunomodulatory action. AM3 is characterized by a protein/polysaccharide fraction that confers high resistance against enzymatic degradation and acidic pH in the stomach (27, 40). The potential beneficial effect of AM3 depends on its ability to reach its targets. However, once in the body AM3 must pass through a series of physiological barriers that can override it. As such, digestive enzymes and/or blood proteases can hydrolize and inactive AM3. However, the disulfide bridges of RicC3 allow AM3 to be a very stable compound, resistant to denaturation, and scarcely modifiable to proteolytic cleavage. This allows its high bioavailability. Furthermore, AM3 is not altered by liver metabolism and does not affect the hepatic bioconjugation system. Therefore, it does not alter the effect of drugs co-administered with AM3 (29, 41, 42). In this way the polysaccharide/protein structure of AM3 remains virtually unchanged and confers high bioavailability. This situation allows high concentrations of AM3 in the bloodstream to be achieved and to generate inactive fragments (29, 41, 42).

**Figure 1:**
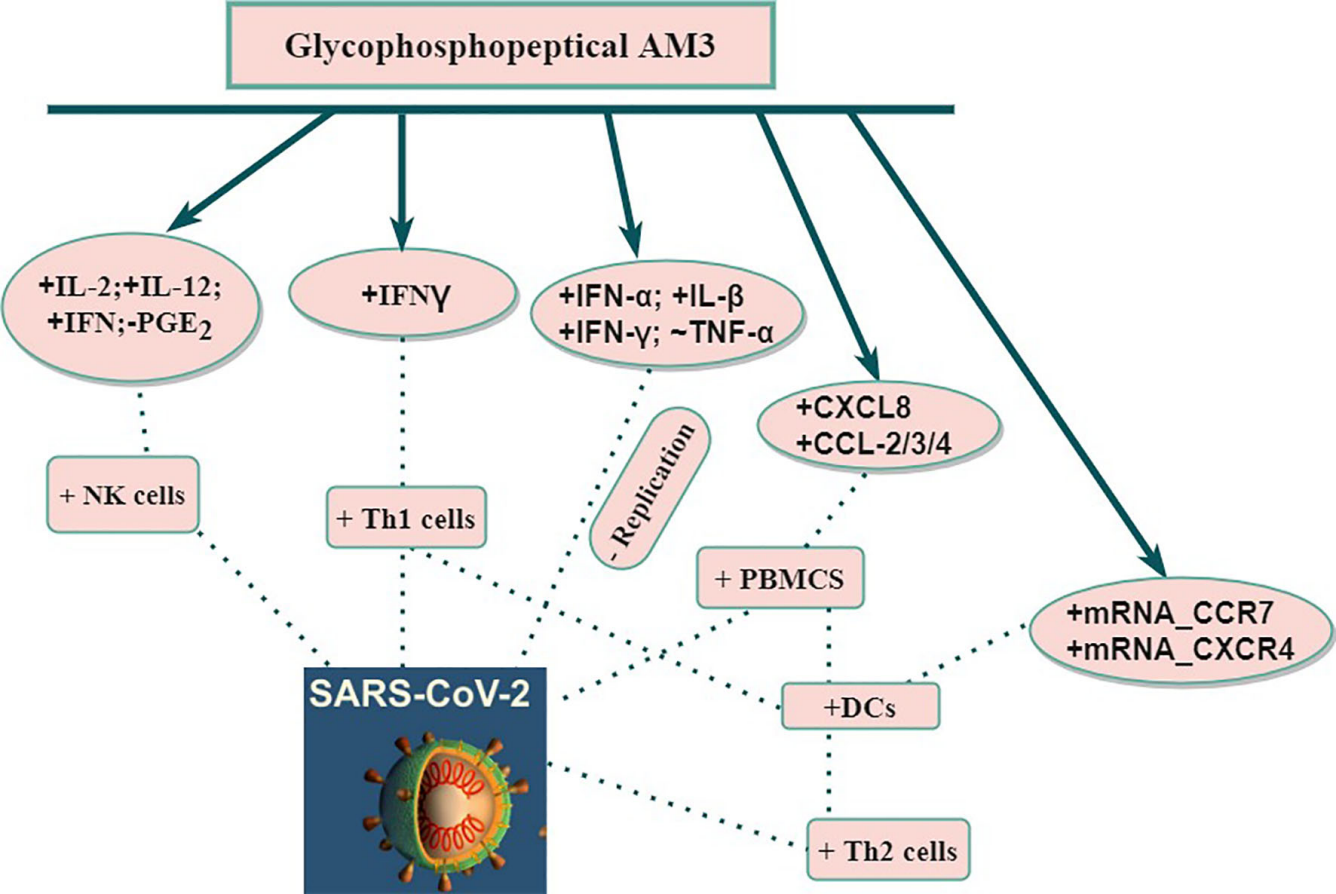
Potential of use AM3 on immune response against SARS-CoV-2. CCL, Chemokine (C-X-C motif); CCR, Chemokine (C-C motif) Receptor; CXCL, Chemokine (C-X-C motif) Ligand; CXCR, Chemokine (C-X-C motif) Receptor; DCs, Dendritic cells; IFN, Interferon; IL, Interleukin; mRNA, Messenger RNA; NK, Natural Killer; PBMCS, Peripheral blood mononuclear cells; PGE, Prostaglandin; SARS-CoV-2, Severe Acute Respiratory Syndrome-2; Th1, T helper 1; Th2, T helper 2; TNF, Tumor Necrosis Factor; +, Stimulation; -, Inhibition; ~, Modulation.

The polysaccharide fraction of AM3 interacts with endogenous mediators associated with intestinal lymphatic tissue. After AM3 polysaccharide has been absorbed and entered into the blood system, it interacts with the circulating dendritic cells (DCs) (37, 43, 44). These immunomodulatory properties are useful in the clinical setting in reducing decompensations and exacerbations in chronic obstructive pulmonary disease (COPD) patients caused by infectious respiratory diseases (viral and bacterial) (24, 45) with no side effects (25–28, 31, 33, 43, 44, 46–49). Thus, AM3 in COPD patients stimulates recovery of T-cell proliferation, restores IFN-γ production, increases the number of natural killer cells (NKC), and restores phagocytic activities. As such, a decrease in the rate of COPD exacerbation was observed in patients (24, 45). Additionally, AM3 was able to spark non-specific immune-system modulation (increase in antibody-producing B lymphocytes) in mice (47) without any alteration of toxicity indicators such as lactate dehydrogenase (LDH) and glutamic oxaloacetic transaminase (GOT) levels. Precautions, however, should be taken in pregnant and hypercalcemic patients (29, 31). Due to the effectiveness of AM3, a prophylactic setting is proposed in certain respiratory infections (like COPD exacerbations without significant toxicity. Therefore, AM3 may have potential use as adjuvant therapy for COVID-19 diseases by way of immune modulation (**Figure 1**).

## Role of AM3 in the SARS-CoV-2 Immune Response

The immune response against viruses consists of two phases. The first is non-specific and starts immediately after the entry of the virus into the host; this is called the innate immune response. The second is far more specific and may take some time to appear this is called the called adaptive or acquired immune response. Overall, the immune response plays a fundamental role in SARS-CoV-2 infection. It is necessary, therefore, to overcome and eliminate the virus but also appears to be responsible for the onset of severe and life-threatening disease. The severe damage to lung tissue that sometimes results from COVID-19 primarily reflects inflammation caused by an exaggerated immune response against the SARS-CoV-2 (50, 51).

### Impact on Innate Immunity

#### AM3 and the Production of Natural Killer Cells

Natural killer cells (NKCs) act *via* extracellular death receptors and by exocytotic release of their content in the form of cytolytic granules. NKCs can eliminate virus-infected cells (44), restrict viral replication (52), and contribute to the early immune responses to viruses (53). Some NKC-activity abnormalities exist among activity in patients with common human respiratory viruses such as influenza virus, respiratory syncytial virus (RSV), probably because they have evaded NKC responses (54).

NKCs have been associated with cytokines such as interferon (IFN), interleukin-2 (IL-2) and interleukin-12 (IL-12) (55). IFN activity allows recruitment of pre-NKCs and enhancement of the cytolytic ability of active NK cells. Further, IL-2 and IL-12 maintain some spontaneous NK cell activity (46, 56). In animal models, AM3 (30 mg/kg/day or 60 mg/kg/day) significantly increased cytotoxic activity of NKCs through a positive induction of IFN and IL-2 (46). In this sense, Rojo et al. (57) administered AM3 (30 mg/kg/day) to several groups of mice 2, 3 or 7 days/week. Each *in vivo* treatment of AM3 improved the immune system’s effector functions by enhancing IL-2 and NKC functions, after 15 days of treatment. Moreover, in mouse models AM3 (150 mg/kg/day) this effect lasted for 4 consecutive days, induced IL-12, IFN production, and boosted NKC, immune responses (58).

On the other hand, AM3 inhibits prostaglandin-synthesizing cells directly or indirectly through an inhibition of the intracellular cyclic adenosine monophosphate (cAMP) (46). Prostaglandins (PGs), especially PGE2, are involved in the efficacious activity of NKCs (59, 60). Although the mechanism is unknown, AM3 probably inhibits the generation of PGE2 and/or blocks the E-type prostanoid receptor 4 (EP4). The activation of the EP4 receptor is necessary for the suppression of NKC function (61).

AM3 supplementation has been applied for diseases of the respiratory system. The clinical effect of AM3 treatment in chronic bronchitis, in which NKC depression occurs, is improving physiological NKC levels (23). NKC cytotoxicity peaked at 2 days after AM3 treatment; it remained elevated above control values for up to 8 days after a regimen of 3g/day of oral supplementation over 60 consecutive days in mice (58). Moreover, in patients with COPD, AM3 was able to stabilize diminished NKC function (24). In one study (24), 60 COPD patients received AM3 during 90 consecutive days at oral doses of 3g/day. The results showed that clear defects in the immunity of COPD patients are counteracted by stimulation of peripheral blood cells, especially polymorphonuclear neutrophils (PMNs), NKC, and monocytes/macrophages (MM) by way of AM3 supplementation (24).

In the case of COVID-19 infections, Demaria et al. (62), have observed a dysfunctional state combined with a decrease in NKCs in the blood and lungs of COVID-19 patients, which suggests that NKCs do not participate in the hyper-inflammatory characteristics of ARDS. Hence, AM3 supplementation may stimulate cytotoxic NKC in COVID-19 patients. This is primarily due to NKC’s crucial role in antiviral immune responses and their contribution to the early immune responses to viruses (53). This could attribute some therapeutic properties to AM3 in COVID-19 patients.

#### AM3 and Cytokines

The use of AM3 has been demonstrated to be effective in modulating cytokines (25, 26, 43, 44). Supplementation of AM3 has been correlated with a significant increase in the production of interleukin-10 (IL-10) and IL-12 cytokines by DCs (43, 44). These results are similar to those described by Lagenkamp et al. (63) in stimulating DCs with LPS. IL-12 is essential as a performance link between innate and adaptive immunity. In addition, IL-12 stimulates the production of IFN-γ in NK and T cells. Finally, IL-12 drives/boost an immune response by means of Type 1 T helper cells (Th1) (64).

IL-10 is a cytokine that develops a primary immune response based on Type 2 T helper cells (Th2). IL-10 also has anti-inflammatory and immunomodulatory properties. Thus, IL-10 is secreted by DCs, T cells, and macrophages (65). IL-10 plays an important role in limiting IL-12 production and downregulation of the inflammatory response (66). Finally, Martín-Vilchez et al. (43) have described that the use of AM3 may stimulate IFN-γ production, which is a Th1-stimulating cytokine, without increasing interleukin-4 (IL-4), which polarizes the immune response with Th2.

IL-4 triggers eosinophilia that accompanies airway inflammation. AM3 generally promotes an increase in IL-12, IFN-γ associated with unaffected IL-4 and stimulates a T-lymphocyte response through Th1 (43, 62). Such results have suggested the application of AM3 as an immunological enhancer of the host response against viral infection and novel adjuvant anti-viral treatment.

COVID-19 has established that the hyperinflammatory response induced by SARS-CoV-2, which contributes to the pathogenesis, is a major cause of disease severity and death in infected patients (67). In the early stages of the infection, a group of cytokines and pro-inflammatory chemokines are expressed including interleukin-1β (IL-1β), IL-2, IL-6, interleukin-8 (IL-8), both IFN-α/β, tumor necrosis factor (TNFα), CCL, CCL3, CCL5, CCL2, and IP-10. In the hyperinflammatory phase, patients with severe COVID-19 exhibit higher levels of IL-2, IL-6, IL-7, IL-10, IP-10, MCP1, TNF-α, macrophage inflammatory protein 1 alpha (MIP1A), and granulocyte-colony stimulating factor (G-CSF) than patients with moderate infections (early inflammatory phase). The fluctuations of these cytokines IL-6, and TNF-α exceed the physiological range (68). Thus, the overproduction of these cytokines and chemokines may contribute to lung damage and potentially fatal respiratory complications. This cytokine storm probably down-regulates innate and adaptive immunity against SARS-CoV-2 infection (69, 70).

There is a relationship between the immune system and the hypothalamus-pituitary-adrenal (HPA) system manifested by the secretion of glucocorticoids and other HPA-derived molecules. In COVID-19, the inflammatory response stimulates an initial acute phase characterized by monocyte/macrophage activation and the expression of pro-inflammatory cytokines such as TNF-α, IL-1 and IL-6 (49, 71). This triggers fever and the production of acute phase proteins of hepatic origin with a potent anti-protease activity that allows attenuation of inflammation and tissue destruction (28).

Brieva et al. (28) have reported that doses of 3, 6 and 9 mg/kg of AM3, dissolved in water for oral administration, stimulated anti-protease activity in hepatocyte cultures. AM3 has a plausible anti-inflammatory effect because it inhibits TNF-α and IL-6 induction by LPS in murine models (27, 28).

In athletes, strenuous exercise (elite competitive sport) has been associated with changes in cytokine production, specifically with increases in plasma concentrations of IL-1, IL-6 and TNF-α to supra-physiological levels (3, 72). The functional and potentially clinical significance of competitive sport-induced alterations in inflammatory cytokines results in generalized inflammation of the body, tissue damage, myalgia, alteration of the immune system (susceptibility to infection), fever, and chronic fatigue (73). These situations somehow coincide with the situations described in COIVD-19 patients. Thus, it is necessary to regulate the immune system as an essential therapeutic objective in inflammatory states such as strenuous exercise and COVID-19 infections. For example, researchers have observed that in elite cyclists, if treated with AM3 at a dose of 3 g/day for periods of 65 and 180 consecutive days, IL-6 and TNF-α were significantly reduced (25). Moreover, a significant increase in TNF-α receptors were observed with respect to the untreated group of AM3 (25, 26). TNF-α receptors have anti-inflammatory properties because they allow binding and neutralizing circulating and membrane-bound TNF-α (26). These immunomodulatory effects are a consequence of the oral administration of AM3 (25–29) and could be applied as a potential therapeutic adjuvant for SARS-CoV-2 treatment.

**Figure 2:**
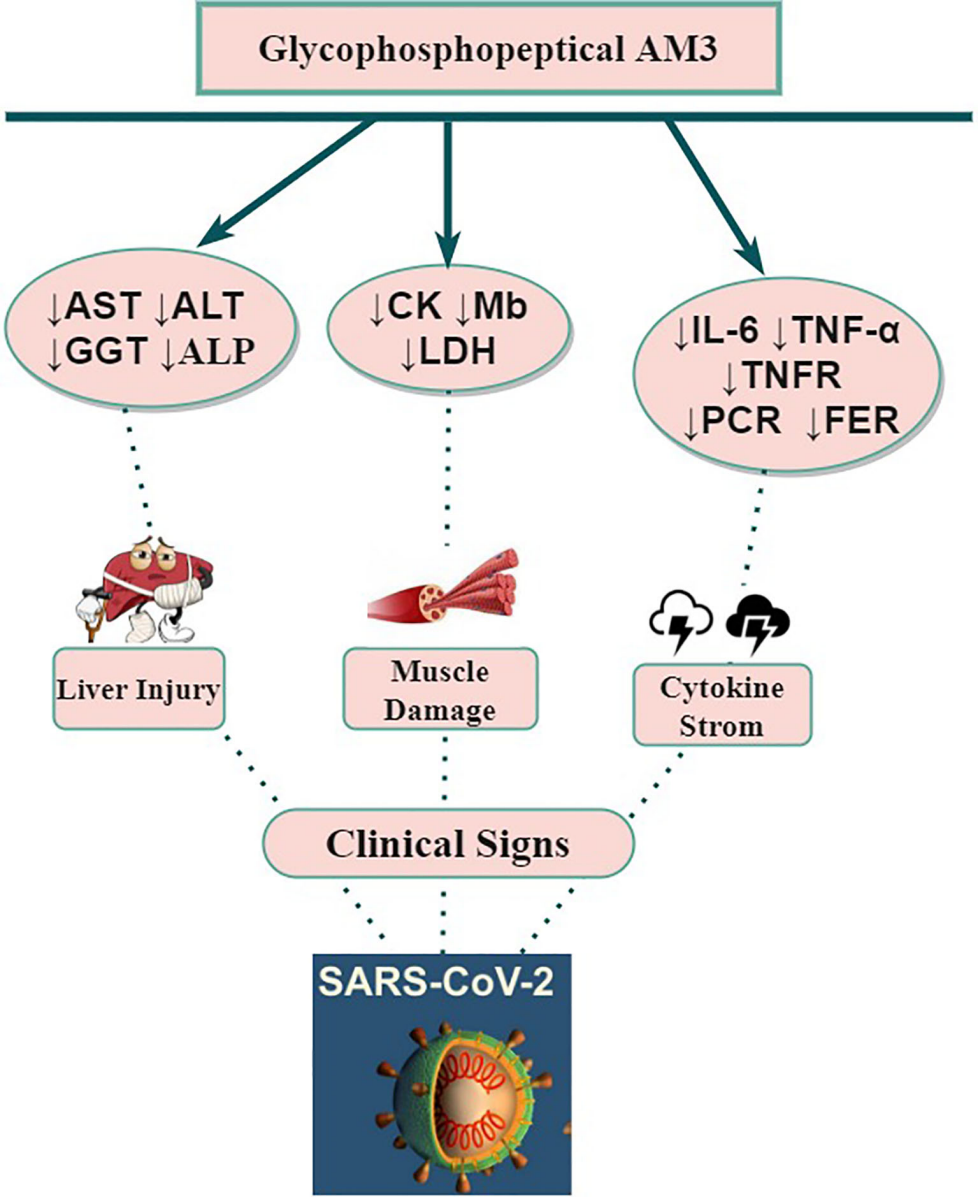
Potential beneficial impact(s) of AM3 supplementation on COVID-19 clinical features and outcomes. ALP, Alkaline Phosphatase; ALT, Aspartate Alanine aminotransferase; AST, Aspartate Aminotransferase; CK, Creatine Kinase; Gamma Glutamyl Transpeptidase; IL, Interleukin; LDH, Lactate Dehydrogenase; Mb, Myoglobin; SARS-CoV-2, Severe Acute Respiratory Syndrome-2; TNF, Tumor Necrosis Factor; TNFR, Tumor Necrosis Factor Receptor; ↓, Decrease.

Alternatively, AM3 increased the plasma levels of corticosterone, which is also stimulated by HPA during the inflammatory process (27). Corticosterone and other glucocorticoids have been described to modulate the expression of mRNA of TNF-α (74). This supports the idea that the AM3 pathway is through direct interaction with HPA to control plasma TNF-α elevation during inflammatory processes (28). The potential modulation of inflammation of AM3 (**Figure 2**) causes a reduction of IL-6 and its ability to neutralize soluble TNF-α or to block TNF receptors from binding to their ligands. These multiple biological activities may attenuate the cytokine storm syndrome which often contribute to the severe pathogenesis of COVID-19.

An alternative to TNF-α hyper-response modulation in COVID-19 infections is the use of anti-TNF-α antibodies (75). However, most drugs that target TNF-α also block bioavailable TNF-α that monocytes and T cells produce, increasing the susceptibility to viral or bacterial coinfection or reinfection (76). AM3 normalizes the TNF-α production (27); in particular, AM3 inhibited TNF-α production by 90% compared to sera from placebo-treated mice. Taken together, these data suggest a regulatory role of AM3 in the response to the increased level of TNF-a production to physiological levels. Although many AM3 studies have demonstrated a high safety profile, its use must be monitored in COVID-19 infections.

On the other hand, the most relevant anti-viral defense element of innate immunity is the action of IFNs to limit and fight against viral infections. IFNs recruit neutrophils, control infection, modulate inflammation and develop the initial containment against invasion by COVID-19 (77, 78). IFN provides a response that would block the spread of the virus and allow the body the time necessary for the generation of a more specific and potent immune response (79, 80).

AM3 is also an immunological response modifier of IFN (46). Moya et al. (46) have observed increases in serum IFN concentrations when AM3 and IFN inducers (Newcastle disease virus (NDV) and/or bacterial lipopolysaccharide (LPS)) were coadministered in BALB/c mice (45). However, investigators observed that AM3 is not an IFN inducer per se. The IFN’s inhibitory effect reflects impaired NDV binding to cell-surface receptors because of viral glycation inhibition that blocks early interaction between the virus and host cells (81). Although, the type of IFN is not clearly specified, the results showed that in early stages AM3 act as a stimulator of NK cytotoxicity through IFN (46). In this sense, Type I IFNs act directly on NK cells to promote their activation, cell cycle entry and cytotoxic function in early stages viral infection (82). This might suggest that the IFN involved was IFN-I (α/β). Moreover, AM3 promotes natural immunity that is related to the induction of endogenous IFN-γ production in animal models when given at doses of 150 mg/kg/day for 4 consecutive days and patients with chronic bronchitis (3g/day during 60 consecutive days) (58). As described above, AM3 could exert different effects on each type of IFNs.

Host cells detect SARS-CoV-2 ribonucleic acid (RNA) in the cell cytosol and activate the IFN synthesis pathway. IFN secretion protein synthesis in infected host cells to prevent the virus from using “cell factories” to produce its proteins and replicate. This IFN activity causes cell death, which enters a process called apoptosis (83).

However, patients with severe COVID-19 infections have a lower expression of IFN-γ related to the decrease and impairment of cluster of differentiation 4+ (CD4+), cluster of differentiation 8+ (CD8+), and NKC (84). A high analytical interleukin-6 (IL-6)/IFN ratio seems to predict severe COVID-19 disease and lung damage due to the cytokine storm (85). In this way, IFN-I levels are decreased (absence of IFN-β; low IFN-α production) which is a clinical feature of severe COVID-19 (81). SARS-CoV structural proteins, envelope proteins (EP), membrane proteins (MP), nucleocapsid phosphoproteins (NP) (86), and in SARS-CoV-2 two non-structural polyprotein (open reading frame [ORF] ORF1a and ORF1b) inhibit the release and secretion of IFN-I (87, 88). The viral proteins block IFN-I signaling by the Janus kinase (JAK) pathway, and consequently, decrease the signal transducer and the activator of signal transducer and activator of transcription protein (STAT1) (86). To solve this situation, the use of AM3 may effectively increase the levels of IFN (46, 58). IFN acts on neighboring cells and there, it activates genes that confer resistance to the infection trough antiviral and immunomodulatory activity which could attribute some therapeutic properties to AM3 against SARS-CoV-2 (83).

#### Interaction Between AM3 Supplementation and Nitic Oxide Production

Nitric oxide (NO) regulates physiological functions in the cardiovascular, nervous, muscular, and immune systems. In the immune system its action is non-specific towards tumor cells, virus or microorganisms, but it has also been associated with mechanisms of tissue damage, as well as in the inhibition of lipid oxidation by the lipoxygenase (LOX) and cyclooxygenase (COX) pathway (89). NO impairs the dissemination of immune cells and inflammatory points by altering cell adhesion and activation (90).

The synthesis of NO through one of the nitric oxide synthase isoforms, inducible Nitric Oxide Synthase (iNOS or NOS-II), is located in the smooth muscle cells and in the monocytes and macrophages. Expression of iNOS occurs in association with the local and/or systemic inflammatory response (91). iNOS is associated with inflammatory diseases of the airways and in the body’s defense against infection. The iNOs generate more than 1,000 times the NO than the other nitric oxide synthase isoforms (eNOS and cNOS) and their production continues for a long period (92). As in the case of COVID-19 patients, their effect can be much more generalized, which can constitute a pathogenic mechanism *via* amplification of the inflammatory response with potentially harmful consequences. NO can enhance the inflammatory response by readily binding to the superoxide anion generating peroxynitrite ions that act directly on inflammatory cells. Elevated concentrations of NO can produce vascular damage in the endothelium causing toxic shock (93). This is similar to other inflammatory conditions, such as rheumatoid arthritis, chronic inflammatory bowel disease and atherosclerosis where excessive NO production by iNOS exacerbates tissue damage (94).

Lung injury caused by COVID-19 often evolves rapidly with ARDS followed by multiple organ failure due to a cytokine storm (79). In this way, bronchial epithelial cells produce NO, associated with overexpression of iNOs, after exposure to pro-inflammatory cytokines such as IL-1β and TNF-α (95). IL-1β and TNF-α will enhance the effects of NO. This process is like other chronic respiratory diseases such as asthma and COPD with a high inflammatory component (96). Dexamethasone is used in COVID-19 patients undergoing invasive mechanical ventilation and/or supplemental oxygen to reduce mortality (97). Dexamethasone acts by inhibiting the nuclear factor κβ (NF-κβ) of epithelial cells which decreases NO production (90). Corticosteroids can significantly benefit pulmonary function in ARDS patients, but they can also have adverse effects, which may have a detrimental impact on long-term outcome (98). AM3 supplementation has no known side effects that compromise other functions of the body that would compromise health (25–28, 31, 33, 43, 44, 46–49) and modulate NO production in mice (32). Specifically, the expression of iNOS inhibition resulted in a significant decrease in serum NO levels after treatment with AM3 (3 mg*Kg) for 6 consecutive days (32). Altogether, these results suggest that AM3 modulates the NO response as well as its possible role in the control of the inflammatory response associated with IL-6 and TNF-α (25, 32). Thus, AM3 could provide an adjuvant therapeutic option in order to reduce levels of dexamethasone doses and its side effects.

### Impact on Adaptative Immunity

#### AM3 Effects on Peripheral Blood Mononuclear Cells

Peripheral blood mononuclear cells (PBMCs) are critical components of the immune system (99). PBMCs include various types of lymphocytes (T cells, B cells, and NKCs), DCs, and monocytes (99).

AM3 and AM5 (active product molecule of AM3) act on PBMCs by stimulating the immune response (48, 49). It inhibits hepatitis B virus (HBV) replication, deoxyribonucleic acid (DNA) synthesis, and viral antigen expression through activation of PBMCs (49). Furthermore, AM3 stimulates secretion of IFN-α, IFN-γ, IL-β and modulate TNF-α (49), which have properties that indirectly control viral infections by PBMCs (71, 74). The SARS-CoV can infect and replicate in peripheral blood mononuclear cells (PBMCs) (100), invasion by SARS-CoV-2 could trigger deregulation of humans PBMCs. In this sense, *in vitro* SARS-CoV-2 infection of human PBMCs revealed they are susceptible cell types (69). Although the precise mechanism of cell invasion is unknown, investigation of apoptotic markers on T lymphocytes and CD147 expression levels could explain the mechanism of invasion and/or replication of SARS-CoV-2 in PBMCs.

Another mechanism that allows indirect AM3-control of viral infections is the activation of human DCs derived from human monocyte-derived dendritic cells (MDDCs) (44). MDDCs have been shown to initiate and maintain responses to pneumonia and lung inflammation, often playing a role in resolution. Their interaction with COVID-19 remains unclear but could be effective (67). MDDCs include the main complex of major histocompatibility (MHC) classes I and II, as well as molecules for co-stimulation (101). AM3 has a potential similar to LPS in the expression of co-stimulatory molecules and MCH in patients infected with HBV that control chronic HBV infection (43).

Although there is no evidence yet of the potential of AM3 against COVID-19, the boost of the immune system *via* PBMCs or MDDCs may help to develop an antiviral immune response in these patients as adjuvant therapeutic approach. Because these immune cell subsets are altered during severe clinical stages of COVID-19 (102). Additionally, with increasing disease severity monocytes and MDDCs are substantially altered in number and function in blood and lungs during COVID- 19 infections (102). In severe and critical stages of COVID-19 infections, levels of neutrophils, lymphocytes (80% of patients present lymphopenia with a significantly alarming decrease between 80-100% of CD4+ and CD8+), and DCs in peripheral blood are reduced (103, 104).

DCs are the principal antigen-presenting cells for T cells involved in the innate and adaptive immune system. They recognize pathogen-related structures and stimulate T-cell activity (105). Dendritic cell-specific intracellular adhesion molecules (ICAM) - dendritic cell-specific ICAM-grabbing non-integrin (DC-SIGN) is a DC-specific adhesion receptor that binds with high affinity to ICAM3 that recognizes high mannose glycan that are present on pathogens (106). DCs are abundant in the lung and have a maximum peak of activity in early and severe stages of COVID-19 infections (79).

DCs infected with SARS-CoV-2 suggest the possible exacerbation of immunopathology. Nonetheless, the possibility of increased recruitment, accelerated maturation, and activation of DCs could combat COVID-19 infection (107, 108). AM3 upregulates C-X-C Motif Chemokine Ligand 8 (CXCL8), C-C Motif Chemokine Ligand (CCMCL), CCL2, CCL3 and CCL4 that are implicated in the conscription and maturation of DCs. DCs must migrate to the lymph nodes, where they will present antigens to the T-lymphocytes to perform their immune function (109). This process depends on the expression of the cc-chemokine receptors 4 (CCR4) and C-X-C chemokine receptor type 7 (CXCR7). CCR7 also regulates migration speed, survival, and differentiation in the CDs (110). Moreover, AM3 and LPS upregulate expressions on messenger RNA (mRNA) for the chemokine receptors CXCR4 and CCR7. Consequently, AM3 might be useful in regulating immune responses in pathophysiological situations that require DCs maturation (43).

SARS-CoV-2 stimulates IL-6 overproduction of alveolar macrophages through the toll-like receptor 4 (TLR4)-mediated NF-κB signaling pathway (111, 112). TLR4 is a receptor within the innate immune system, which recognizes pathogen-associated molecular patterns (PAMPs) of SARS-CoV-2 (3). AM3 is a TLR4 agonist (43). In the presence of AM3, TLR4/NF-kβ receptor would preferentially trigger p38 mitogen-activated protein kinase (p38MAPK) as a consequence of NF-kβ activation, which plays an essential capacity in the stimulation of immature DCs (43). Which would displace at least partially IL-6 production of alveolar macrophages mediated the TLR4/NF-kβ receptor. In other words, AM3 binds to the receptor TLR4/NF-kβ and prevents the SARS-CoV-2 from developing its effect. By increasing the AM3 concentrations, the effect could be achieved (competitive agonist). This would reduce IL-6 production and dampen exacerbated inflammation that leads to acute lung injury (113).

The diverse immunopathology caused by the COVID-19 infection could be a consequence of the interaction of the SARS-CoV-2 Spike (S) protein with the DC-SIGN receptor in respiratory DCs. DC-SIGN has been shown to mediate the binding of the SARS-CoV S protein to human DC with absorption into the endosome, followed by polarization of the endosome and presentation of the virus at an infectious synapse. Similarly, Human Immunodeficiency Virus (HIV) establishes the infectious synapse between T cells and DCs mediated by DC-SIGN (103). For this reason, a strategy to control the infection by COVID-19 could result in inhibiting initial stages of infection and dissemination of pathogens in the DCs through DC-SIGN, as it has been demonstrated by the oral AM3 administration (44) AM3 acts, dose dependently, on MDDCs by blocking the adhesion of pathogens such as Candida spp., Aspergillus spp. and Leishmania spp. by the interaction of AM3 on the activity of DC-SIGN of MDDCs. In relation to HIV, AM3 inhibits the function of DC-SIGN as a regulator of cell adhesion by blocking its binding to ICAM-3, which suppresses the binding of HIV gp120 to DC-SIGN in DCs. AM3 overrides the capacity of cells that express DC-SIGN to trap and transmit HIV viruses with replicative activity (44). Thus, it is expected that the application of AM3 would be beneficial against immunopathology caused by the COVID-19 infection because AM3 directly influences pathogen recognition by DCs altering functional capabilities of DC-SIGN. This could establish a therapeutic approach to early-stage SARS-CoV-2 infection.

#### Might AM3 be a Potential Adjuvant in the Vaccination Against SARS-CoV-2?

SARS-CoV-2 vaccines come in several types: whole-virus, subunit, attenuated, viral vectors, and nucleic acids; most are based on protein subunits (114). As of May 2021, fifteen vaccines have been approved for at least one country and thirty-three are in phase III in clinical trials (115). There are three vaccines approved by the FDA and [Pfizer-BioNTech, Moderna, and Janssen Pharmaceutical Companies of Johnson & Johnson (J&J’s)] and another has been approved by the European Medicines Agency (EMA) in the European Union (Oxford/AstraZeneca). Other vaccines, such as the Novavax vaccine is expected to be approved in the next few months (116, 117).

BionTech/Pfizer have jointly developed BNT162b2 as an mRNA vaccine against SARS-CoV-2. The mRNA causes the host-cell to produce S-antigen proteins to stimulate an immune response (118). The efficacy has been demonstrated in clinical trials among participants with and without evidence of prior SARS-CoV-2 infection. Subjects who received the full, two-dose vaccine regime showed an approximately 95% efficacy rate after a median follow-period up of 2 months (119).

The mRNA-1273 vaccine, Moderna’s COVID-19 vaccine, is a messenger RNA vaccine (118). It has been shown to have an efficacy of 94.1%, also based on a median follow-up of two months. The high efficacy was maintained across all age groups (above 18 years) and was not affected by gender or ethnicity (120). AstraZeneca’s COVID-19 (AZD1222) vaccine (C19VAZ), formerly known as ChAdOx1 nCoV-19, is made from a virus (ChAdOx1), a weakened version of a common cold virus (adenovirus). Genetic material has been added to the ChAdOx1 construct, which is used to make the SARS-CoV-2 coronavirus proteins called Spike (S) glycoprotein (118). The efficacy demonstrated in clinical trials in participants who received the full vaccine series (2 doses), regardless of the interval between doses, was 63.1%, based on a median follow-up of 80 days, but with a tendency to increase the longer gap between doses (121). J&J’s vaccine (Ad26.COV2.S) is based on incompetently replicating recombinant adenovirus serotype 26 (Ad26) vector, encoding a stabilized, full-length SARS-CoV-2 spike protein (118). J&J’s vaccine was 66.3% effective in clinical trials in preventing confirmed COVID-19 disease in persons with no evidence of previous infection two weeks after receiving the vaccine. Individuals achieved maximum possible protection within two weeks after vaccination (122). Although Novavax’s vaccine (NVX-CoV2373) has not been approved yet, its Phase 3 clinical trial conducted in in the United Kingdom (UK) has demonstrated promising results with 89.3% of efficacy (118), even against the UK and South African variants (123).

AM3 has shown to be a useful adjuvant in hepatitis B vaccination in healthy people who previously did not develop Hepatitis B surface antigen (HBsAg) > 10 IU/ml titers in response to recombinant hepatitis B vaccine (34). Also, oral administration of AM3 (3 g/d), in patients with advanced renal disease and undergoing hemodialysis who did not respond to hepatitis B vaccination, for 30 consecutive days beginning 15 days before the first dose of vaccine maintained protective titers until six months after the final dose of vaccine. But not in the control group (33). In addition, the percentage of patients with high response (anti-HBsAg > 100 IU/L) and medium anti-HBsAg titers in the AM3 group was significantly higher than in the placebo group (33). This demonstrates that AM3 is a safe and easily tolerated oral agent that boosts long-term serological immunity to hepatitis B by developing prolonged protective anti-HBsAg titers that allow for long-term serological immunity after vaccination.

It is believed that most of the new COVID-19 vaccines may be low in immunogenicity in humans (114). Relative to previous studies (33, 34), administration of the AM3 immunomodulatory may result in a clear and prolonged antibody response that flows to SARS-CoV-2 vaccination. The significant increase in the percentage of patients with protective anti-HBsAg antibody titers and an increase in the rate of naive patients who maintain a protective response after six months of follow-up, induced by the adjuvant use of AM3 (33, 34), could support AM3 as an adjuvant to COVID-19 vaccination.

Although the mechanism is unknown, the stimulation and conservation of antibody titers observed are likely due to the establishment of long-term immune memory due to the influence of enhanced cell-mediated immunity function (124). The cell-mediated immunity function is considered the main mechanism of protecting and eliminating intracellular infectious agents, especially viral infections (125).

## Effect of AM3 on Biochemical Muscular Damage Markers

Muscle damage includes: 1) increased muscle proteins in the blood creatine kinase (CK), lactate dehydrogenase (LDH), and myoglobin (Mb); 2) decreased force generation; 3) capacity inflammatory cell infiltration; 4) disruption of Z-disks (delineate the lateral borders of sarcomeres which are the smallest functional units in skeletal muscle) and cell membrane damage (126, 127). Alterations in the immune system after strenuous exercise of athletes have as a consequence pathological changes in tissues comparable to other diseases such as bacterial sepsis or viral infections (128). In athletes, the inflammatory response is associated with significantly increased muscle proteins such as CK, Mb and LDH in blood (127).

As COVID-19 has demonstrated that cytokine release syndrome (CRS) produces an uncontrolled and overwhelming release of pro-inflammatory and inflammatory facilitators. The cytokine concentration was increased in COVID-19 patients and can be used as a predictive factor of disease severity in patients with COVID-19 (129). This situation produces breakdown of muscular fiber and connective tissue (108). Ultrastructural damage of muscle tissue is a potential complication arising from COVID-19-associated inflammatory cytokine production and release causing severe muscle injury (130). Muscle fibers contain proteolytic enzymes that, upon injury, are released and initiate degradation of lipid and protein structures of the injured cell. The rapid breakdown of damaged muscle fibers and connective tissue is accompanied by diffusion of intracellular components into the plasma (127). Patients with muscle weakness, myalgia, muscle atrophy, myositis, and rhabdomyolysis have been observed with some of the symptoms most commonly reported by patients with COVID-19. All of them had elevated serum levels of CK, Mb, LDH, and elevated serum levels of CRP and FER (131).

In a study conducted on basketball players (30), the placebo group experienced significant increases in CK, Mb and LDH after 30 days of exercise practice. The supplementation of AM3 in the experimental group, not only inhibited these changes, but also resulted in a significant decrease from baseline in serum concentrations of CK, Mb and LDH. AM3 prevents the changes of muscular damage biomarkers after exercise in elite athletes (29). Cordova et al. (25, 31) have reported that AM3 supplementation of 5 g/day during 6 weeks reduce plasma levels of enzyme activities associated with muscle damage. Therefore, the use of AM3 in patients with COVID-19 could be an effective therapeutic alternative to attenuate muscle damage (**Figure 2**) but also in post-COVID-19 recovery.

The muscular biomarkers that may provide information on the time course of COVID-19 are based on clinical findings associated with SARS, MERS and other viral respiratory infections (132). Variations in the biomarkers of muscle damage such as CK, Mb and LDH may help predict the course of COVID-19-associated pneumonia. Severe infections produce tissue damage and organ failure that play a more important role in SARS-CoV-2 through cytokines and release of CK, Mb and LDH (132–135). In this way, the biochemical results of muscle damage found in SARS-CoV-2 infected patients show that the muscle damage marker CK was elevated in 14% of COVID-19 patients (134). Mb elevations were more pronounced in severe patients admitted to ICU *vs.* non-severe hospitalized patients with COVID-19 (104). With respect to LDH, a 6-fold and 16-fold increase in LDH increased the likelihood of severe symptoms and increases in mortality and morbidity in COVID-19 patients (132, 135).

## Conclusion

AM3, a polysaccharide/protein compound, is a naturally occurring oral immunomodulatory compound. The AESAN categorizes AM3 as “Food Supplement” with regulatory effects on the immune system. AM3 has shown a wide range of regulatory effects on innate and adaptive immunity in experimental and clinical models of inflammation and disease. AM3 is an effector of immune-cell activity involved in response to SARS-CoV-2. AM3 activates the NKC production, positively modulates IFN secretion, promotes PBMC activity, and reduces inflammatory cytokine production. AM3 also reduces muscle, hepatic and pulmonary damage, decreases circulating NO levels. To date, no study has been reported neither side effects nor toxicity of the use of AM3. However, in future clinical trials, a pharmacovigilance appendix would be desirable in infections such as COVID-19 that frequently involve hyper-inflammatory responses. Clinical studies of AM3 in COVID-19 patients are needed to assess whether AM3 may be a preventive and therapeutic strategy in COVID-19 infections. Also, AM3 may be a useful adjuvant to SARS-CoV-2 vaccination.

## Author Contributions

Conceptualization, DF-L and CF-L. Methodology, DF-L, CF-L, and JM-A. Writing—original draft preparation, DF-L and CF-L. Writing—review and editing, DA, JM-A, JGH, and MG-G. Visualization, CF-L, DPA, JG, and MG-G. Supervision, DF-L. All authors contributed to the article and approved the submitted version.

## Funding

Call for expressions of interest for the funding of research projects on SARS-CoV-2 and COVID-19 disease by the FONDO-COVID19 n° 07.04.467804.74011.0 within the framework of Royal Decree Law 8/2020 of 17 March on extraordinary urgent measures to deal with the economic and social impact of COVID-19. Financed by the FEDER and the Junta of Castilla-Leon, Spain.

## Conflict of Interest

The authors declare that the research was conducted in the absence of any commercial or financial relationships that could be construed as a potential conflict of interest.
